# Independent Dutch Validation Study of CP-GEP (Merlin Assay) for the Prediction of Nodal Metastasis and Long-Term Outcome in Patients with Primary Cutaneous Melanoma

**DOI:** 10.1245/s10434-025-18928-9

**Published:** 2025-12-18

**Authors:** Lisanne P. Zijlker, Zahra Verwer, Bart A. van der Wiel, Anke M. J. Kuijpers, Michel W. J. M. Wouters, Winan J. van Houdt, Alexander C. J. van Akkooi

**Affiliations:** 1 Netherlands Cancer Institute–Antoni van Leeuwenhoek (NKI-AVL), Amsterdam, The Netherlands; 2 Leiden University Medical Center, Leiden, The Netherlands; 3 Melanoma Institute Australia, The University of Sydney, Sydney, Australia; 4 Faculty of Medicine and Health, The University of Sydney, Sydney, Australia; 5 Department of Melanoma and Surgical Oncology, Institute of Academic Surgery, Royal Prince Alfred Hospital, Sydney, Australia

## Abstract

**Background:**

Currently, sentinel lymph node biopsy (SLNB) is considered standard staging for patients with stage IB–II melanomas. Owing to recent systemic therapy advances, SLNB is once again being scrutinized. The aim of the current study was to validate the diagnostic accuracy of the clinicopathological gene expression profile (CP-GEP) for risk of sentinel node metastases and recurrence.

**Patients and Methods:**

Samples and clinical data of 252 patients with newly diagnosed clinical stage I/II melanoma between 2007 and 2015 were obtained. CP-GEP included eight target genes associated with tumor development (i.e., *MLANA, GDF15, CXCL8, LOXL4, TGFBR1, ITGB3, PLAT*, and *SERPINE2*) and two housekeeping genes in combination with clinicopathological variables, age, and Breslow thickness.

**Results:**

The median age was 57 years old, and 51.4% were female. The median Breslow thickness was 1.8 mm, and 53 patients (21.8%) had a positive SLNB. The median follow-up was 91 months. CP-GEP identified 68 (28%) patients as CP-GEP low risk and 175 (72%) as high risk. The sensitivity of CP-GEP was 92.5%, specificity was 33.7%, positive predictive value (PPV) was 28.0%, and negative predictive value (NPV) was 94.1% for all (T1–T4). The CP-GEP had the best performance in T1, with an NPV of 95.2% and an SLNB reduction rate (RR) of 80.8%. In the pT1b–pT2a melanomas with an NPV of 93.3% and a SLNB RR of 45.5% in terms of long-term survival, the 5-year recurrence-free survival (RFS) of CP-GEP low risk was 89.6% versus 76.8% for CP-GEP high-risk patients.

**Conclusions:**

CP-GEP demonstrated good prognostic performance, particularly for patients with pT1b–pT2a melanoma and thus could be considered a noninvasive alternative for a SLNB.

**Supplementary Information:**

The online version contains supplementary material available at 10.1245/s10434-025-18928-9.

Currently, sentinel lymph node biopsy (SLNB) is still considered the most appropriate tool and standard of care to stage patients with melanoma, who present with newly diagnosed stage IB–II melanomas.^1^ With the developments in systemic therapy, specifically the shift toward earlier stages of disease, the use of the minimally invasive, but nonetheless invasive, SLNB is once again being scrutinized.^2^

The majority (~75–85%) of all eligible patients that undergo SLNB do not harbor any metastatic disease.^3,4^ Despite the prognostic power of SLNB, the absence of the SLNB metastasis does not exclude the risk of recurrence or death, especially not for stage II (T2b–4bN0) patients.^5^ Therefore, any improved prognostication, particularly if it would involve a less or even a noninvasive procedure, would be considered to be an improvement over current practice. Gene expression profiling (GEP) of the primary tumor is one of the tools that is being studied in oncology. Already, in breast cancer, this has been established as a method to identify patients, on the basis of their primary tumor in the absence of nodal involvement, who might benefit from (adjuvant) systemic therapy versus those who are unlikely to benefit.^6^

GEP has been shown to be able to identify either melanoma patients who are likely to have metastases within their SLNB and also to predict long-term disease outcome for early stage (I–II) melanoma patients.^7–12^ The Merlin^TM^ Assay is a clinically available test that uses the clinicopathological (CP)-GEP model, combining clinicopathological variables and gene expression of the primary melanoma to identify patients who have a low risk for SN metastasis and subsequently a low risk of recurrence too.^9^

The aim of the current study was to validate the diagnostic accuracy of the CP-GEP on an independent cohort from the Netherlands Cancer Institute.

## Patients and Methods

Patients with newly diagnosed clinical stage I/II melanoma, who presented at the Netherlands Cancer Institute (NKI), Amsterdam, the Netherlands, between 2007 and 2015, were eligible for this current study. Data were prospectively collected in an institutional database and were retrospectively analyzed. The data cutoff for follow-up was January 2022. This study was approved by the institutional research board (IRB20-033) and conducted in accordance with national and local ethical guidelines.

SLNB was performed in accordance with common recommendations.^13^ In brief, patients underwent lymphatic mapping via lymphoscintigraphy + single-photon emission computed tomography (SPECT-CT) to identify the draining sentinel nodes. Either the same day or the next day, the SLNB was performed. Initially, blue dye was routinely injected, just prior to incision, but later on, blue dye might be omitted if nodes were easily retrievable with the gamma probe alone.^14^ After surgical removal, the node(s) were processed according to the updated EORTC Melanoma Group pathology protocol.^15^

The Merlin Assay^®^ is a registered conformité européenne - in vitro diagnostic (CE-IVD), clinically available test that uses the CP-GEP model. The CP-GEP model combines the expression of eight genes (*SERPINE2*, *GDF15*, *ITGB3*, *CXCL8*, *LOXL4*, *TGFBR1*, *PLAT*, and *MLANA*) and two housekeeping genes (*RLP0* and *ACTB*) by quantitative polymerase chain reaction (qPCR) using in ΔCt method, with the clinicopathological variables age and Breslow thickness to obtain a binary output: CP-GEP low risk or CP-GEP high risk. RNA was extracted from formalin-fixed paraffin-embedded (FFPE) blocks from each primary tumor from the NKI-AVL biobank. In total, 50 microns of primary FFPE material was used for GEP testing as previously described.^9^ Only the initial diagnostic biopsy tissue (either excisional, shave, or punch) was used, and no wide local excision material was used.

The SLNB prediction performance of CP-GEP model was determined by calculating its sensitivity, specificity, positive predictive value (PPV), negative predictive value (NPV), SLNB reduction rate (RR), and corresponding 95% Clopper–Pearson confidence intervals (CIs).^16^ The SLNB RR is calculated as the proportion of patients that are classified by the CP-GEP model as low risk and may forgo the SLNB on the basis of the test outcome: SLNB RR = (CP-GEP low risk)/(CP-GEP low risk + CP-GEP high risk).^17^ All performance measures were stratified by T-category according to American Joint Committee on Cancer (AJCC) eighth-edition staging system.^5^ Statistical analyses were performed using R software (4.2.2; R Foundation for Statistical Computing, Vienna, Austria), and *p*-values lower than 0.05 were considered statistically significant. Patient characteristics were summarized using the gtsummary package in R (version 1.7.0).

The prognostic ability of CP-GEP was evaluated using Kaplan–Meier curves. The clinical end points were recurrence-free survival (RFS), distant metastasis-free survival (DMFS), and melanoma-specific survival (MSS). The hazard ratio (HR) was calculated with a 95% CI using a Cox proportional hazards regression model. A Wald *p*-value lower than 0.05 (two-sided) was considered statistically significant. The follow-up evaluation was capped at 5 years. Patients experiencing an event beyond this duration were censored at the 5-year mark. The median follow-up period was determined using the reverse Kaplan–Meier estimator prodlim in R (version 2019.11.13).

## Results

**Fig. 1:**
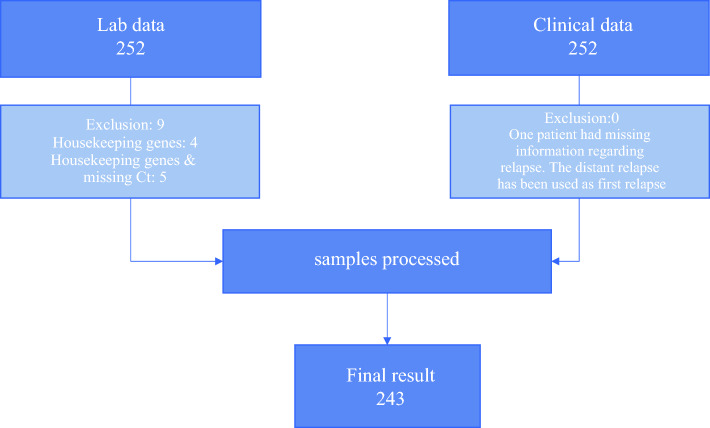
Consort diagram

A total of 252 patients’ samples and clinical data were submitted. In nine patients, the CP-GEP was unable to be performed, and in *n* = 4, this was due to housekeeping gene Ct’s being too high (1.6%) and *n* = 5 due to housekeeping and target gene Ct’s too high and/or missing (2.0%) (Fig. 1). The final analysis was performed on the remaining 243 cases. The median age was 57 years old (interquartile range (IQR) 46–67), and 51.4% were female. The median Breslow thickness was 1.8 mm (IQR 1.3–2.95), and 53 patients demonstrated involvement of the SLN (21.8%). Over half of the patients were diagnosed with pT2 tumor, of which, 44.9% were pT2a. The median follow-up for RFS was 91 months (IQR 67–116), for DMFS was 92 months (IQR 68–116), and for MSS was 92 months (IQR 69–116). Table 1 summarizes the baseline patient and tumor characteristics.

**Table 1:** Patient and tumor clinicopathologic characteristics. n (%) or median (interquartile range)

Variable	Level	AVL-NKI	CP-GEP low risk	CP-GEP high risk
*n*		243 (100%)	68 (28.0%)	175 (72.0%)
Gender	Female	125 (51.4%)	32 (47.1%)	93 (53.1%)
	Male	118 (48.6%)	36 (52.9%)	82 (46.9%)
Age (years)	Median (IQR)	57 (46, 67)	59 (51, 67)	55 (44, 66)
Breslow depth (mm)	Median (IQR)	1.80 (1.30, 2.95)	1.20 (1.00, 1.50)	2.10 (1.50, 3.25)
Ulceration	Absent	185 (76.1%)	66 (97.1%)	119 (68.0%)
	Present	57 (23.5%)	1 (1.5%)	56 (32.0%)
	Unknown	1 (0.4%)	1 (1.5%)	0 (0.0%)
SLNB outcome	Negative	190 (78.2%)	64 (94.1%)	126 (72.0%)
	Positive	53 (21.8%)	4 (5.9%)	49 (28.0%)
Stages	IA	24 (9.9%)	20 (29.4%)	4 (2.3%)
	IB	91 (37.4%)	38 (55.9%)	53 (30.3%)
	IIA	36 (14.8%)	5 (7.4%)	31 (17.7%)
	IIB	28 (11.5%)	0 (0.0%)	28 (16.0%)
	IIC	10 (4.1%)	0 (0.0%)	10 (5.7%)
	III	53 (21.8%)	4 (5.9%)	49 (28.0%)
	Unknown	1 (0.4%)	1 (1.5%)	0 (0.0%)
T categories	T1	0 (0.0%)	0 (0.0%)	0 (0.0%)
	T1a	4 (1.6%)	3 (4.4%)	1 (0.6%)
	T1b	22 (9.1%)	18 (26.5%)	4 (2.3%)
	T2	1 (0.4%)	1 (1.5%)	0 (0.0%)
	T2a	109 (44.9%)	41 (60.3%)	68 (38.9%)
	T2b	15 (6.2%)	1 (1.5%)	14 (8.0%)
	T3	0 (0.0%)	0 (0.0%)	0 (0.0%)
	T3a	39 (16.0%)	4 (5.9%)	35 (20.0%)
	T3b	27 (11.1%)	0 (0.0%)	27 (15.4%)
	T4	0 (0.0%)	0 (0.0%)	0 (0.0%)
	T4a	12 (4.9%)	0 (0.0%)	12 (6.9%)
	T4b	14 (5.8%)	0 (0.0%)	14 (8.0%)
Biopsy location	Head neck	44 (18.1%)	12 (17.6%)	32 (18.3%)
	Trunk	110 (45.3%)	37 (54.4%)	73 (41.7%)
	Extremity	87 (36.4%)	18 (26.5%)	66 (37.7%)
	Acral undefined	5 (2.1%)	1 (1.5%)	4 (2.3%)
Histologic type	Superficial spreading	169 (69.5%)	51 (75.0%)	118 (67.4%)
	Nodular	61 (25.1%)	10 (14.7%)	51 (29.1%)
	Desmoplastic	1 (0.4%)	1 (1.5%)	0 (0.0%)
	Lentigo maligna	6 (2.5%)	4 (5.9%)	2 (1.1%)
	Acral lentiginous	3 (1.2%)	2 (2.9%)	1 (0.6%)
	Spitzoid	1 (0.4%)	0 (0.0%)	1 (0.6%)
	Other	2 (0.8%)	0 (0.0%)	2 (1.1%)
Clark level	2	5 (2.1%)	4 (5.9%)	1 (0.6%)
	3	58 (24.3%)	27 (39.7%)	32 (18.3%)
	4	120 (50.2%)	22 (32.4%)	100 (57.1%)
	5	17 (7.0%)	2 (2.9%)	15 (8.6%)
	Unknown	40 (16.5%)	13 (19.1%)	27 (15.4%)
Lymphovascular invasions	Absent	114 (46.9%)	30 (44.1%)	84 (48.0%)
	Present	12 (4.9%)	1 (1.5%)	11 (6.3%)
	Unknown	117 (48.1%)	37 (54.4%)	80 (45.7%)
Mitotic rate categorical	Absent	19 (7.8%)	13 (19.1%)	6 (3.4%)
	Present	150 (61.7%)	32 (47.1%)	118 (67.4%)
	Unknown	74 (30.5%)	23 (33.8%)	51 (29.1%)

**Fig. 2:**
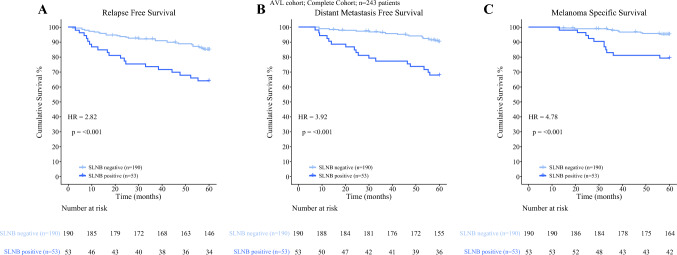
**A** Kaplan–Meier estimated relapse-free survival (RFS), **B** distant metastasis-free survival (DMFS) and **C** melanoma-specific survival (MSS) for SLNB positive and negative patients

For all patients, the 5-year recurrence-free survival (RFS) was 80.4% (95% CI 74.8–84.9%), the 5-year distant metastasis-free survival (DMFS) was 85.3% (95% CI: 80.1–89.2%), and the 5-year melanoma specific survival (MSS) was 91.6% (95% CI 87.3–94.5%). When stratifying for SLNB status, the 5-year RFS was 85.0% (95% CI: 79.0–89.4%) for SLNB-negative versus 64.2% (95% CI 49.7–75.4%) for SLNB-positive patients (Fig. 2). Table 2 summarizes the RFS according to AJCC staging.

**Table 2:** Summarizes the RFS according to different categories

	*N*	Event RFS	5-years RFS, 95%CI
Complete cohort	243	47	**80.4** (74.8–84.9)
SLNB negative	190	28	**85.0** (79.0–89.4)
SLNB positive	53	19	**64.2** (49.7–75.4)
CP-GEP low risk	68	7	**89.6** (79.5 – 94.9)
CP-GEP high risk	175	40	**76.8** (69.8 – 82.4)
IB–IIA	127	12	**90.2** (83.4–94.3)
Stage IA	24	1	**95.8** (73.9–99.4)
Stage IB	91	8	**91.0** (82.7–95.4)
Stage IIA	36	4	**88.2** (71.5–95.4)
Stage IIB	28	12	**56.7** (36.5–72.7)
Stage IIC	10	3	**70.0** (32.9–89.2)
Stage III	53	19	**64.2** (49.7–75.4)
Stage NA	1	0	**NA**

Bold font was used to emphasize the main outcome

CP-GEP risk stratification was performed in 243 patients with a SLNB positivity rate of 21.8%; of these patients, 68 (28%) were risk-stratified as CP-GEP low risk and 175 (72%) as CP-GEP high risk. The sensitivity of CP-GEP to predict SLNB metastases was 92.5%, the specificity was 33.7%, the PPV was 28.0%, and the NPV was 94.1% for all comers (T1–T4). CP-GEP had four false-negative patients, of whom three did not relapse. CP-GEP had the best performance in T1 with an NPV 95.2% and SLNB reduction rate (RR) of 80.8% and in the pT1b–pT2a melanomas with an NPV of 93.3% and a SLNB RR of 45.5% (for details, see Table 3), and this was also true for patients > 65 years old (Supplementary Table 1).

**Table 3:** CP-GEP performance in NPV and SLNB RR

Patient subset	*N*	SLNB positivity rate	Specificity	Sensitivity	PPV	NPV	TP	TN	FP	FN	SLNB reduction rate
T1–T2	151	16.6 (11–23.5)	47.6 (38.7–56.7)	84 (63.9–95.5)	24.1 (15.6–34.5)	**93.8** (84.8–98.3)	21	60	66	4	**42.4** (34.4–50.7)
T1–T3	217	21.2 (16–27.2)	37.4 (30.2–45.1)	91.3 (79.2–97.6)	28.2 (21.1–36.1)	**94.1** (85.6–98.4)	42	64	107	4	**31.3** (25.2–38)
T1–T4	243	21.8 (16.8–27.5)	33.7 (27–40.9)	92.5 (81.8–97.9)	28 (21.5–35.3)	**94.1** (85.6–98.4)	49	64	126	4	**28** (22.4–34.1)
T1b–T2a	132	15.2 (9.5–22.4)	50 (40.4–59.6)	80 (56.3–94.3)	22.2 (13.3–33.6)	**93.3** (83.8–98.2)	16	56	56	4	**45.5** (36.8–54.3)

T1	26	7.7 (0.9–25.1)	83.3 (62.6–95.3)	50 (1.3–98.7)	20 (0.5–71.6)	**95.2** (76.2–99.9)	1	20	4	1	**80.8** (60.6–93.4)
T2	125	18.4 (12–26.3)	39.2 (29.7–49.4)	87 (66.4–97.2)	24.4 (15.6–35.1)	**93** (80.9–98.5)	20	40	62	3	**34.4** (26.1–43.4)
T3	66	31.8 (20.9–44.4)	8.9 (2.5–21.2)	100 (83.9–100)	33.9 (22.3–47)	**100** (39.8–100)	21	4	41	0	**6.1** (1.7–14.8)
T4	26	26.9 (11.6–47.8)	0 (0–17.6)	100 (59–100)	26.9 (11.6–47.8)	**NA**	7	0	19	0	**0** (0–13.2)
**T1b**	22	9.1 (1.1–29.2)	85 (62.1–96.8)	50 (1.3–98.7)	25 (0.6–80.6)	**94.4** (72.7–99.9)	1	17	3	1	**81.8** (59.7–94.8)
**T2a**	109	16.5 (10.1–24.8)	41.8 (31.5–52.6)	83.3 (58.6–96.4)	22.1 (12.9–33.8)	**92.7** (80.1–98.5)	15	38	53	3	**37.6** (28.5-47.4)

Bold font was used to emphasize the main outcome

**Fig. 3:**
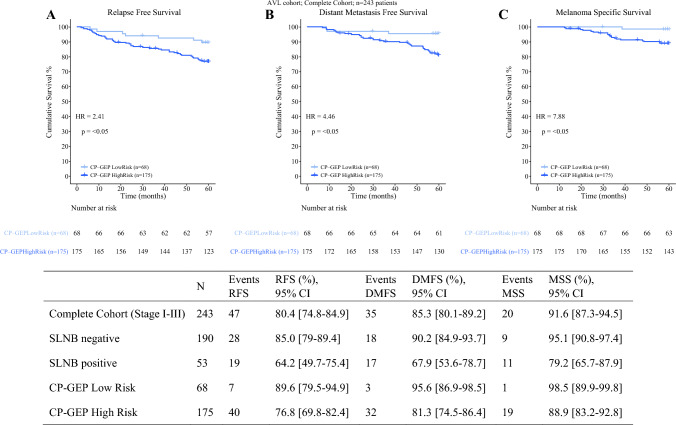
**A** Kaplan–Meier estimated relapse-free survival (RFS), **B** distant metastasis-free survival (DMFS) and **C** melanoma-specific survival (MSS) for CP-GEP low risk versus high risk

**Fig. 4:**
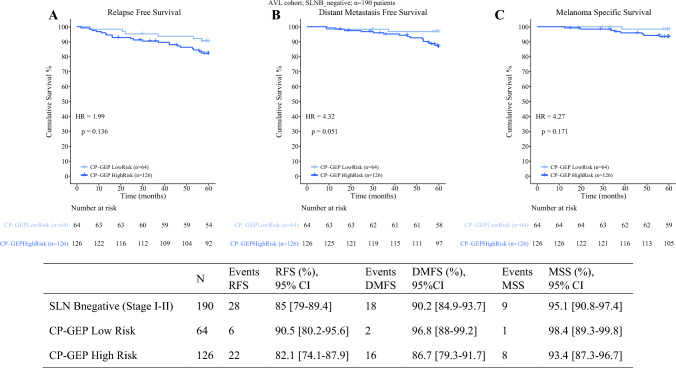
**A** Kaplan–Meier estimated relapse-free survival (RFS), **B** distant metastasis-free survival (DMFS) and **C** melanoma-specific survival (MSS) for SLNB-negative patients risk stratified according to CP-GEP low risk versus high risk

In terms of long-term survival, the 5-year RFS of CP-GEP low risk was 89.6% (95% CI 79.5–94.9) versus 76.8% (95% CI 69.8–82.4) for CP-GEP high-risk patients (Fig. 3). The 5-year DMFS and MSS were 95.6% (95% CI 86.9–98.5) and 98.5% (95% CI 89.9–99.8) for CP-GEP low-risk and 81.3% (95% CI 74.5–86.4) and 88.9% (95% CI 83.2–92.8) for CP-GEP high-risk patients. Considering all patients underwent upfront SLNB, CP-GEP captured 40 out of 47 reported relapses after SLNB (HR of 2.41), 32 out of 35 distant metastasis (HR = 4.46), and 19 out of 20 melanoma deaths (HR = 7.88). Zooming into SLNB-negative patients only, the 5-year RFS of CP-GEP low risk was 90.5% (95% CI 80.2–95.6) versus 82.1% (95% CI 74.1–87.9) for CP-GEP high-risk patients (Fig. 4). The 5-year DMFS was 96.8% (95% CI 88.0–99.2) versus 86.7% (95% CI 79.3–91.7) for CP-GEP low-risk and CP-GEP high-risk patients, respectively. The 5-year MSS was 98.4% (95% CI 89.3–99.8) for CP-GEP low risk versus 93.4% (95% CI 87.3–96.7) for CP-GEP high risk.

Kaplan–Meier-estimated RFS, DMFS, and MSS for substages IA, IB, IB–IIA, IIA, IIB, IIC, and III are provided in the Supplementary Materials.

## Discussion

This large, retrospective, single-institution validation cohort demonstrated good diagnostic accuracy of CP-GEP with respect to the prediction of SLNB metastasis and prognostication of patients with clinical stage I–II melanoma, particularly patients with pT1b–pT2a melanomas, older than 65 years of age and with a negative SLNB.

The CP-GEP used in this study has been previously validated in other independent cohorts. When we compare the results from the current study with the other previously published validation studies, we noticed the current results are very much in line with previously published series. For example, the sensitivity in this study of 92.5% is comparable to other series using the same CP-GEP test at 91–94%.^7,12,18–20^ Similarly, this was seen for the specificity of 33.7% in the current study as compared with others between 23% and 45%.^12,18–20^ PPV was 28.0% and NPV was 94.1% as compared with rates between 15 and 31% and 90% and 97% by others for the same CP-GEP test, respectively.^12,18–20^

Another key observation is that the CP-GEP test performed particularly well for the pT1 melanomas and to a lesser extent for the pT1b–2a melanomas, which is particularly a matter of debate on whether or not to offer a SLNB in the first place, as many (~90–95%) of those patients do not harbor any metastatic disease in their SLNB.^21–25^ Let alone, if pT1b–pT2a patients would then also harbor a significant SLNB tumor burden (> 1 mm in diameter) that would consider them patients with high-risk IIIA melanoma and eligible for adjuvant systemic therapy.^26–29^ Clearly, CP-GEP could be both a noninvasive alternative as well as a cost-effective substitute to a surgical SLNB, which requires surgery (hospital admission, surgical and nursing staff, consumables, etc.) imaging (lymphoscintigraphy), management of surgical complications (pain relief medication, antibiotics, wound care), and more, such as extensive pathological examination.

Another aspect to consider is that we are comparing CP-GEP with SLNB as the “gold standard;” however, SLNB has been reported to have its own limitations. SLNB is known to incur a false negative (FN) percentage of 0–3%;^30^ however, this is not the correct way of calculating the false negative rate (FNR). The FN rate should be calculated as the amount of FN divided by the amount of true positives (TP) plus FN (FN/TP + FN) × 100%.^31^ If it is calculated as such, the FNR for SLNB in melanoma has been reported to be between 9% and 21%.^32^ Hypothetically, this would mean that CP-GEP might be a more accurate risk-predictor than SLNB. Comparing the survival curves of SLNB and CP-GEP might give circumstantial evidence to this point. SLNB positive versus negative has a greater discriminative power (85.0% versus 64.2%; Δ = 20.8%) when compared with CP-GEP low risk versus high risk (89.6% versus 76.8% Δ = 12.8%). However, the 5-year CP-GEP low risk had superior overall survival (OS) rates versus SLNB negative (89.6% versus 85.0%), suggesting CP-GEP low risk is more accurate in determining low risk (potentially owing to a higher rate of false negative SLNB).

We have recently seen the first prospective trial, the Merlin-001 study (NCT04759781), present its data.^33^ Merlin-001 aimed to establish the predictive capability of CP-GEP to identify patients with primary cutaneous melanoma who can safely forego SLNB. The study screened a total of 2141 patients from nine study sites, with 1846 undergoing a SLNB. Indications for SLNB were pT1b–pT3b clinically N0 patients or pT1a patients with adverse features (age < 40 years, tumor mitotic rate > 2 mm^2^ or lymphovascular invasions).^33^ The assay success rate was 97.7%, and 17.6% of patients had a positive SLNB. Low risk CP-GEP had a 6.4% SLNB positivity rate for stage IB melanoma, and this was 6.2% for stage IB ≥ 65 years of age.^33^

Clearly, the current study has limitations. First and foremost, it is a single institutional retrospective study, which carries the obvious risk of selection and reporting bias for the cases that were used. Moreover, the study was conducted in a population of fair Western European ancestry patients, which is not unlike previously studied populations and not for example in other geographical locations, such as Australia and New Zealand, which might incur different genetic mutation patterns compared with the North American and European experiences.

Finally, 26/243 patients (10.7%) that were included had pT4 tumors, which were all high risk. Previous publications have often excluded these patients for this reason. Of the 47 recurrences, 15 were found in stage IIB/C patients. Including these patients might have influenced the observed results. However, first of all, it is valuable to observe this in a real-world cohort and realize that CP-GEP is not of benefit for these patients. Secondly, since it was only 10% of the total sample size, the influence on the observed outcomes is somewhat limited.

Despite these limitations, we believe the current study adds to build the current literature on the utility of CP-GEP in patients with melanoma, particularly with respect to the consistency found in the current study for important diagnostic performance criteria, such as sensitivity, specificity, PPV, and NPV, being very similar with previous observations in other cohorts. CP-GEP seems to be best positioned for patients with pT1b–pT2a melanoma.

Future research might, despite the relatively low specificity, test foregoing surgical SLNB staging for low-risk patients, thereby reducing surgical morbidity and costs, particularly for the pT1b–pT2a cohorts. Although the specificity is relatively low at 33.7%, it would still mean a significant reduction of surgical SLNB procedures.

Other avenues might explore offering adjuvant systemic therapy to high-risk stage IB/IIA patients, despite a negative SLNB. Importantly, SLNB may have a therapeutic value for SLNB-positive patients, as it is often the only involved lymph node, and removal may render these patients disease-free forever, as was seen in MSLT-1 with a 5-year disease-free survival (DFS) of 71.3%.^3^ Moreover, MSLT-2 demonstrated that 11.5% of completion lymph node dissection (CLND) specimen contained additional disease, which means SLNB is a very effective method of controlling the disease in the nodal field.^34^ This was confirmed in a study by Crystal et al., which demonstrated that 80% of SLNB-positive patients will not recur in the nodal field after SLNB.^35^

Finally, any future in which patients are managed with a strategy that foregoes SLNB but offers CP-GEP to determine adjuvant systemic therapy eligibility will need to be prospectively studied. All of this is under the presumption that adjuvant systemic therapy will persist, which is currently a matter of debate, owing to the lack of overall survival benefit being observed so far, as well as the more current neoadjuvant systemic therapy advances.^36–38^

## Conclusions

This CP-GEP demonstrated good predictive and prognostic performance in an independent validation cohort, particularly for patients with pT1b–pT2a melanoma, and thus should be considered added value to current standard of care practice.

## Supplementary Information

Below is the link to the electronic supplementary material.

Supplementary file1 (PDF 331 KB)

Supplementary file1 (DOCX 19 KB)
