# Supplementary material for: Independent Dutch Validation Study of CP-GEP (Merlin Assay) for the Prediction of Nodal Metastasis and Long-Term Outcome in Patients with Primary Cutaneous Melanoma
Source: Ann Surg Oncol. 2025 Dec 18;33(5):3991–9. doi: 10.1245/s10434-025-18928-9 (PMC13083332; doi:10.1245/s10434-025-18928-9)
Supplement: Supplementary file 1 — Supplementary file1 (PDF 331 KB) [file 10434_2025_18928_MOESM1_ESM.pdf]

Suppl Fig 1

Survival stratified by CP-GEP Stage IA

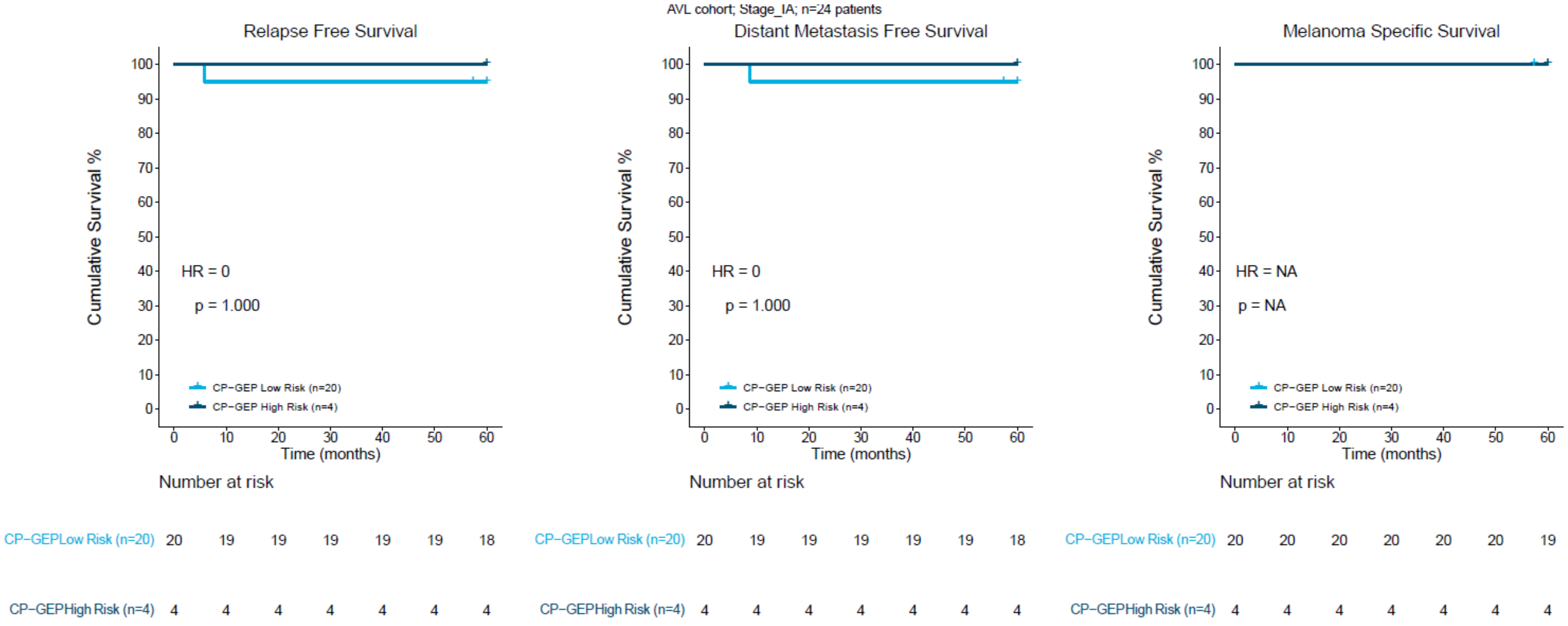

	N	Events RFS	5-years RFS, 95%CI		Events DMFS	5-years DMFS, 95%CI		Events MSS	5-years MSS	95%CI
**Stage IA**	24	1	95.8	[73.9-99.4]	1	95.8	[73.9-99.4]	0	100	[100-100]
**CP-GEP Low risk**	20	1	95	[69.5-99.3]	1	95	[69.5-99.3]	0	100	[100-100]
**CP-GEP High risk**	4	0	100	[100-100]	0	100	[100-100]	0	100	[100-100]

Suppl Fig 2

Survival stratified by CP-GEP Stage IB

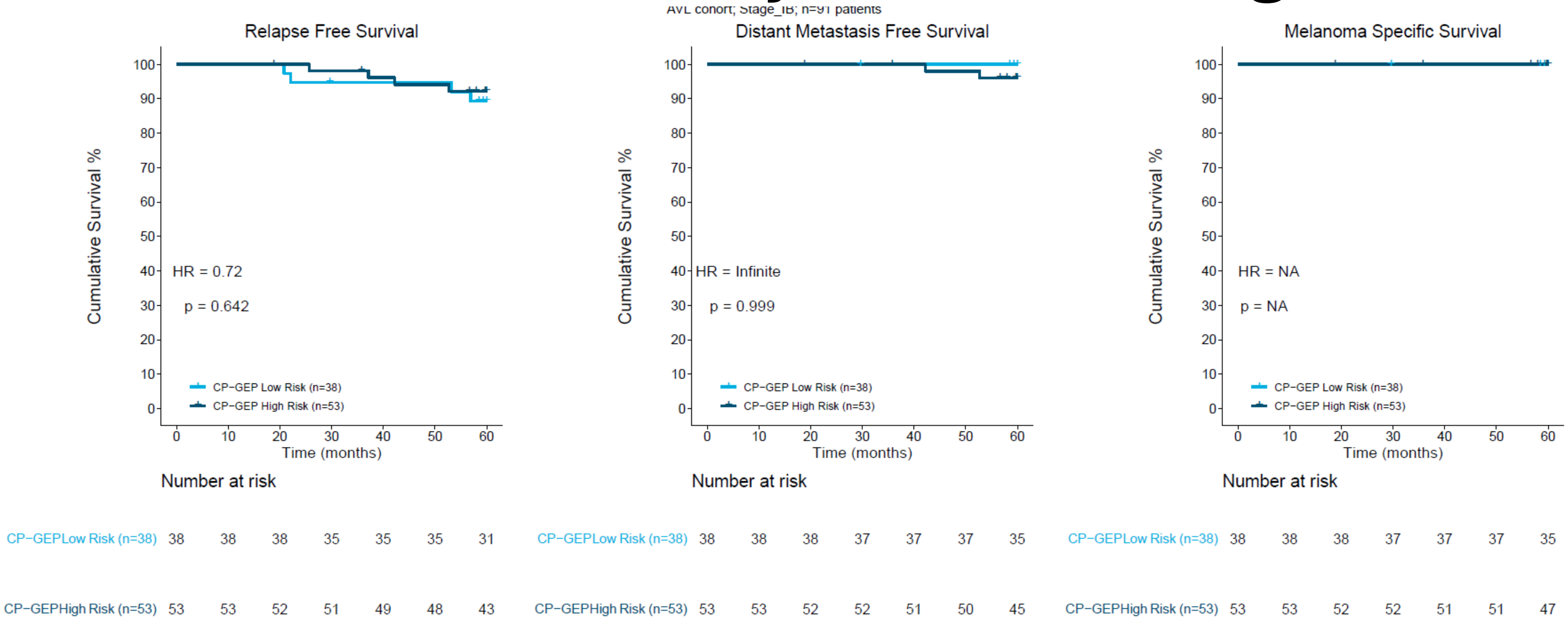

	N	Events RFS	5-years RFS, 95%CI		Events DMFS	5-years DMFS, 95%CI		Events MSS	5-years MSS	95%CI
**Stage IB**	91	8	91	[82.7-95.4]	2	97.7	[91.2-99.4]	0	100	[100-100]
**CP-GEP Low risk**	38	4	89.3	[74-95.9]	0	100	[100-100]	0	100	[100-100]
**CP-GEP High risk**	53	4	92.2	[80.5-97]	2	96.1	[85.2-99]	0	100	[100-100]

Suppl Fig 3

Survival stratified by CP-GEP Stage IB-IIA

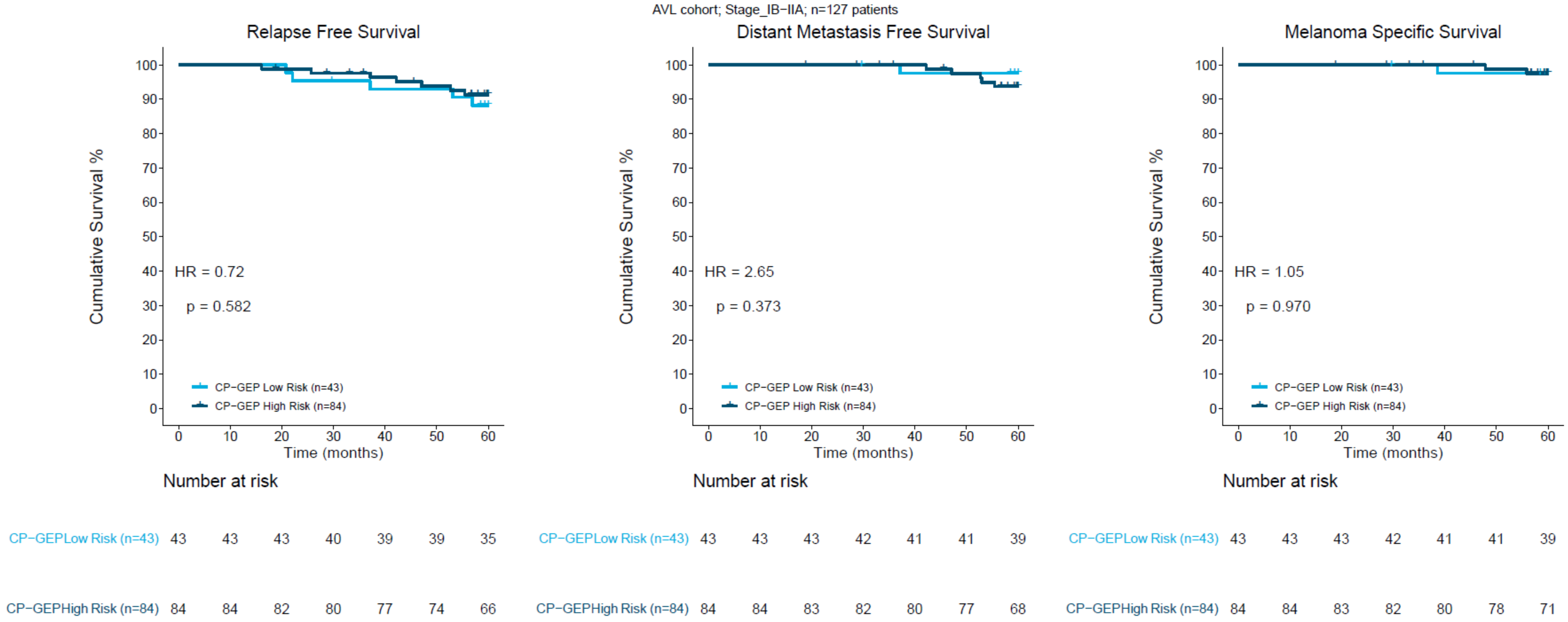

	N	Events RFS	5-years RFS, 95%CI		Events DMFS	5-years DMFS, 95%CI		Events MSS	5-years MSS	95%CI
**Stage IB-IIA**	127	12	90.2	[83.4-94.3]	6	95.1	[89.3-97.7]	6	95.1	[89.3-97.7]
**CP-GEP Low risk**	43	5	88.2	[73.9-94.9]	1	97.6	[84.3-99.7]	1	97.6	[84.3-99.7]
**CP-GEP High risk**	84	7	91.3	[82.6-95.8]	5	93.7	[85.5-97.3]	5	93.7	[85.5-97.3]

Suppl Fig 4

Survival stratified by CP-GEP Stage IIA

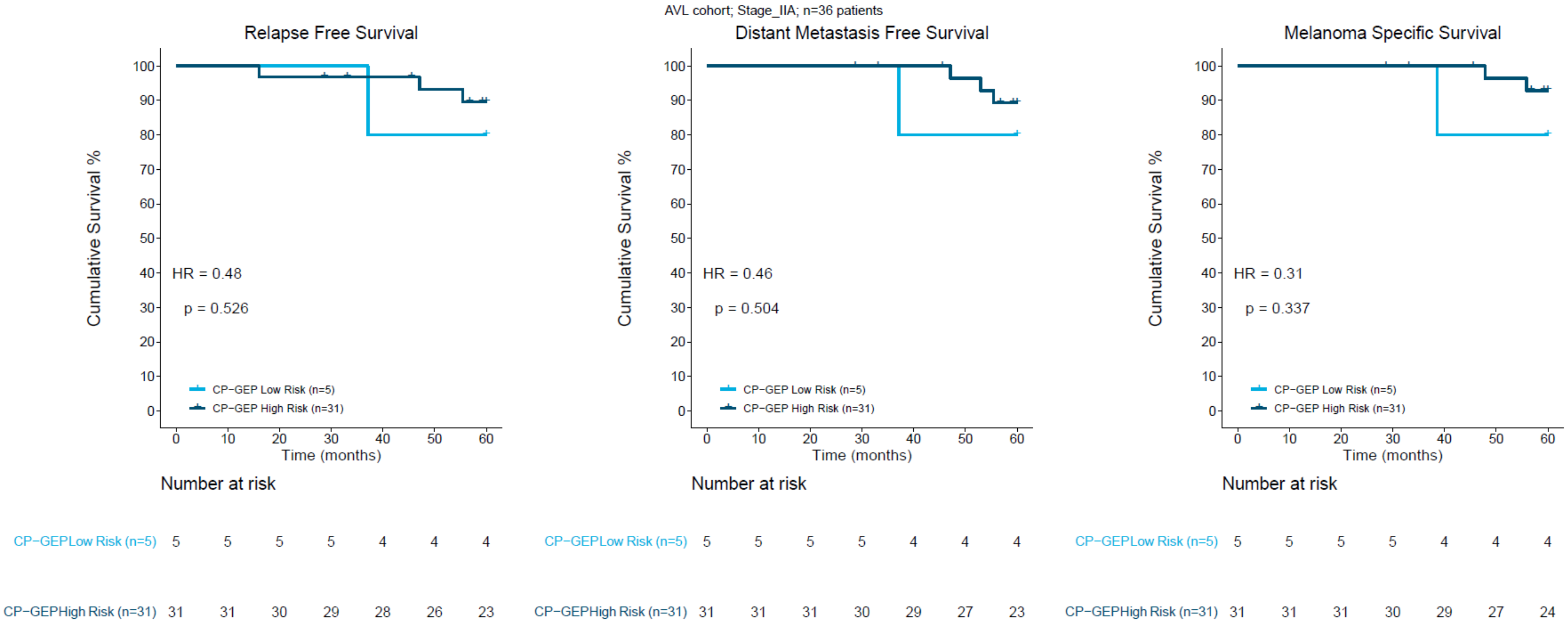

	N	Events RFS	5-years RFS, 95%CI		Events DMFS	5-years DMFS, 95%CI		Events MSS	5-years MSS	95%CI
**Stage IIA**	36	4	88.2	[71.5-95.4]	4	88	[71-95.3]	3	91	[74.6-97]
**CP-GEP Low risk**	5	1	80	[20.4-96.9]	1	80	[20.4-96.9]	1	80	[20.4-96.9]
**CP-GEP High risk**	31	3	89.6	[71.1-96.5]	3	89.3	[70.4-96.4]	2	92.9	[74.3-98.2]

Suppl Fig 5

Survival stratified by CP-GEP Stage IIB

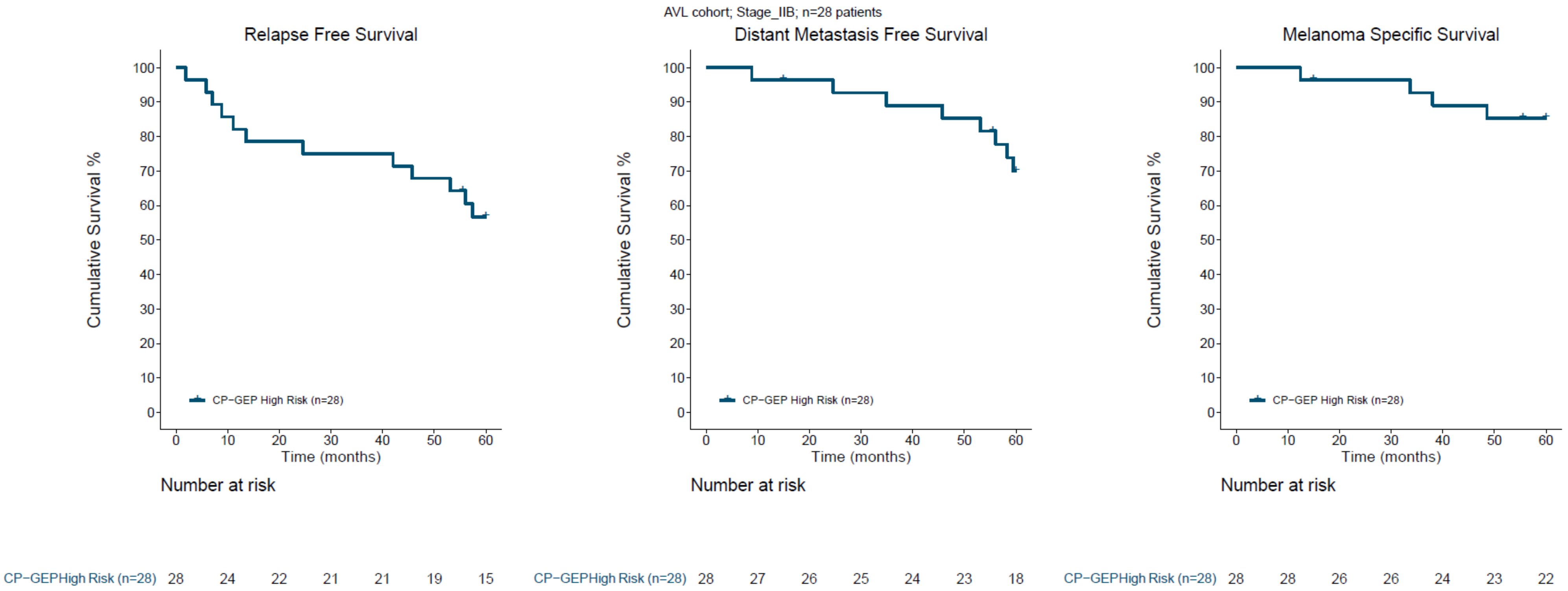

	N	Events RFS	5-years RFS, 95%CI		Events DMFS	5-years DMFS, 95%CI		Events MSS	5-years MSS	95%CI
**Stage IIB**	28	12	56.7	[36.5-72.7]	8	69.9	[48.7-83.7]	4	85.3	[65.4-94.2]
**CP-GEP Low risk**	0	NA	NA	NA	NA	NA	NA	NA	NA	NA
**CP-GEP High risk**	28	12	56.7	[36.5-72.7]	8	69.9	[48.7-83.7]	4	85.3	[65.4-94.2]

Suppl Fig 6

Survival stratified by CP-GEP Stage IIC

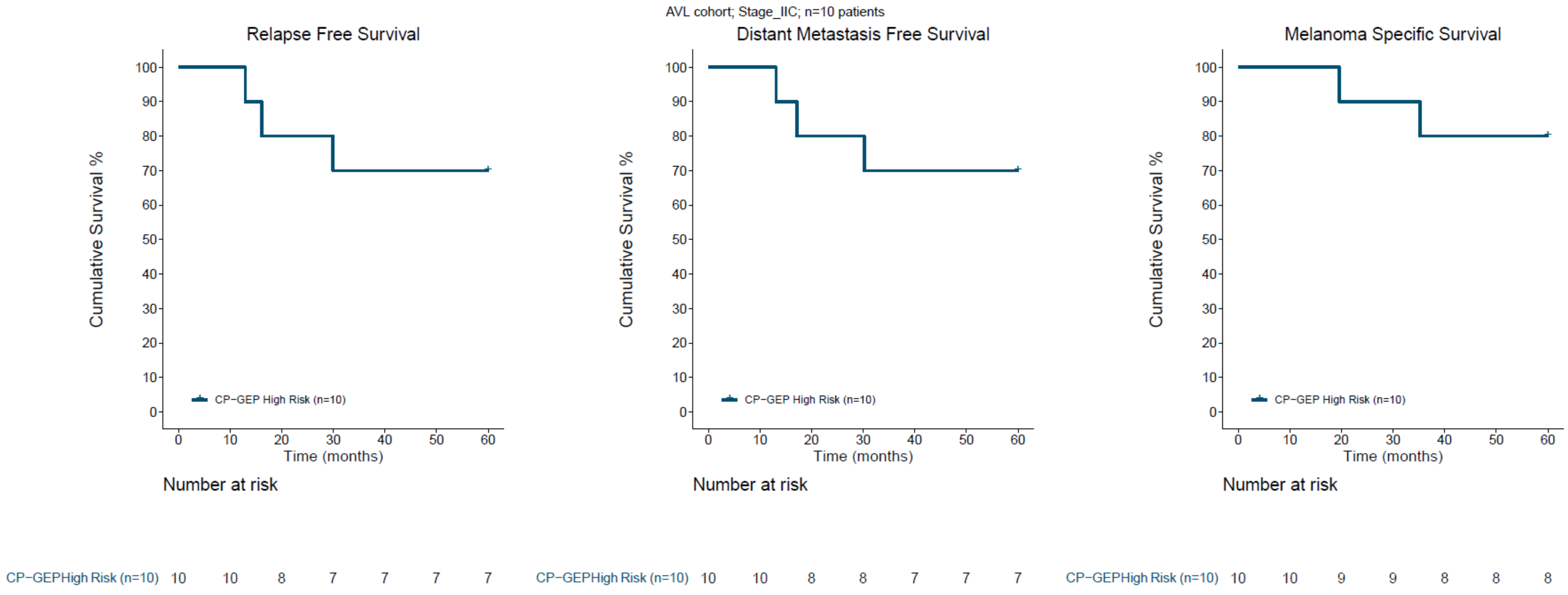

	N	Events RFS	5-years RFS, 95%CI		Events DMFS	5-years DMFS, 95%CI		Events MSS	5-years MSS	95%CI
**Stage IIC**	10	3	70	[32.9-89.2]	3	70	[32.9-89.2]	2	80	[40.9-94.6]
**CP-GEP Low risk**	0	NA	NA	NA	NA	NA	NA	NA	NA	NA
**CP-GEP High risk**	10	3	70	[32.9-89.2]	3	70	[32.9-89.2]	2	80	[40.9-94.6]

Suppl Fig 7

Survival stratified by CP-GEP Stage III

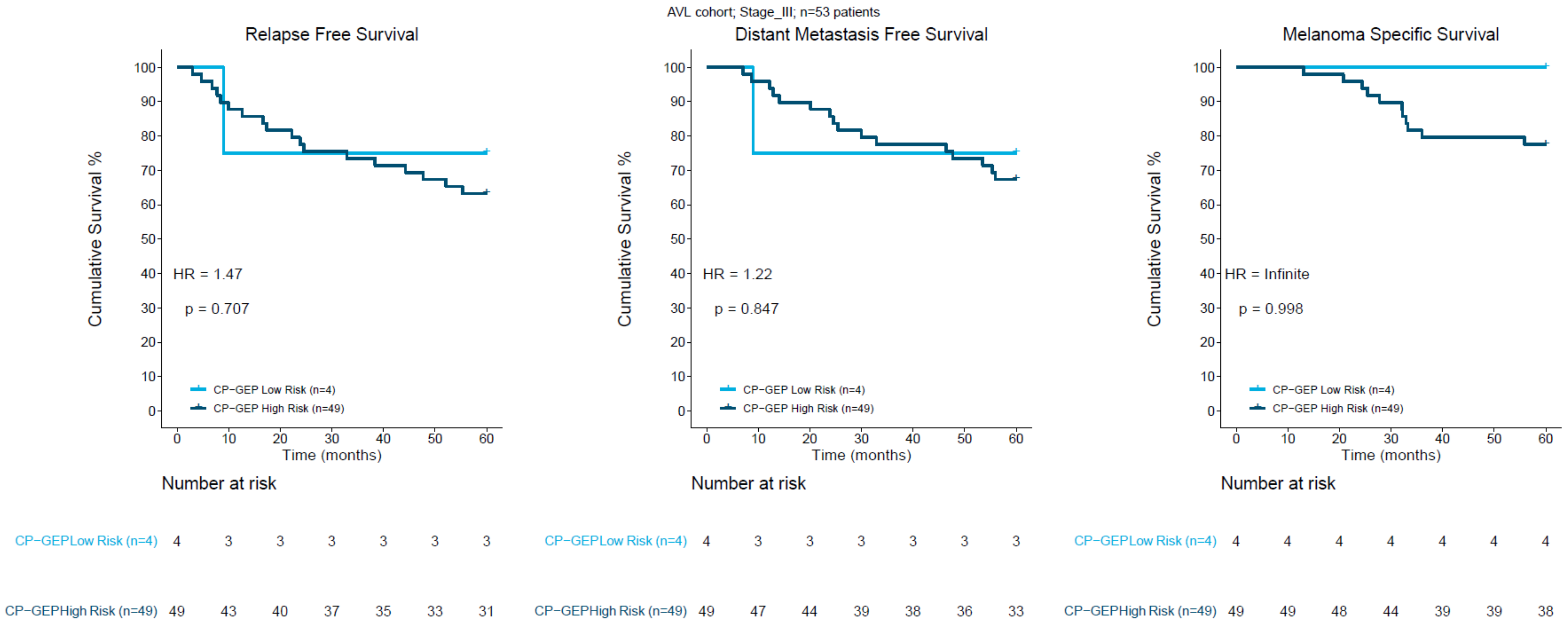

	N	Events RFS	5-years RFS, 95%CI		Events DMFS	5-years DMFS, 95%CI		Events MSS	5-years MSS	95%CI
**Stage III**	53	19	64.2	[49.7-75.4]	17	67.9	[53.6-78.7]	11	79.2	[65.7-87.9]
**CP-GEP Low risk**	4	1	75	[12.8-96.1]	1	75	[12.8-96.1]	0	100	[100-100]
**CP-GEP High risk**	49	18	63.3	[48.2-75]	16	67.3	[52.3-78.6]	11	77.6	[63.1-86.9]
