# Supplementary material for: Independent Dutch Validation Study of CP-GEP (Merlin Assay) for the Prediction of Nodal Metastasis and Long-Term Outcome in Patients with Primary Cutaneous Melanoma
Source: Ann Surg Oncol. 2025 Dec 18;33(5):3991–9. doi: 10.1245/s10434-025-18928-9 (PMC13083332; doi:10.1245/s10434-025-18928-9)
Supplement: Supplementary file 2 — Supplementary file1 (DOCX 19 KB) [file 10434_2025_18928_MOESM2_ESM.docx]

Supplementary table 1: CP-GEP performance for patients >65 years old

| **Patient Subset** | **N** | **SLNB positivity rate** | **Specificity** | **Sensitivity** | **PPV** | **NPV** | **TP** | **TN** | **FP** | **FN** | **SLNB reduction rate** |
| --- | --- | --- | --- | --- | --- | --- | --- | --- | --- | --- | --- |
| T1-T2 | 39 | 12.8 (4.3-27.4) | 55.9 (37.9-72.8) | 60 (14.7-94.7) | 16.7 (3.6-41.4) | **90.5** (69.6-98.8) | 3 | 19 | 15 | 2 | **53.8** (37.2-69.9) |
| T1-T3 | 61 | 14.8 (7-26.2) | 42.3 (28.7-56.8) | 77.8 (40-97.2) | 18.9 (8-35.2) | **91.7** (73-99) | 7 | 22 | 30 | 2 | **39.3** (27.1-52.7) |
| T1-T4 | 73 | 13.7 (6.8-23.8) | 34.9 (23.3-48) | 80 (44.4-97.5) | 16.3 (7.3-29.7) | **91.7** (73-99) | 8 | 22 | 41 | 2 | **32.9** (22.3-44.9) |
